# Facilitators and barriers to implementing lifestyle intervention programme to prevent cognitive decline

**DOI:** 10.1093/eurpub/ckab087

**Published:** 2021-08-27

**Authors:** Jenni Kulmala, Anna Rosenberg, Tiia Ngandu, Katri Hemiö, Tarja Tenkula, Arja Hyytiä, Minna Vienola, Minna Huhtamäki-Kuoppala, Anneli Saarinen, Saana Korkki, Tiina Laatikainen, Alina Solomon, Miia Kivipelto

**Affiliations:** 1 Faculty of Social Sciences (Health Sciences) and Gerontology Research Center (GEREC), Tampere University, Tampere, Finland; 2 Department of Public Health and Welfare, Population Health Unit, Finnish Institute for Health and Welfare, Helsinki, Finland; 3 Department of Neurobiology, Care Sciences and Society, Division of Clinical Geriatrics, Center for Alzheimer Research, Karolinska Institutet, Stockholm, Sweden; 4 Institute of Clinical Medicine/Neurology, University of Eastern Finland, Kuopio, Finland; 5 Southern Ostrobothnia Central Hospital, Seinäjoki, Finland; 6 Härmä Rehabilitation Center, Ylihärmä, Finland; 7 South Ostrobothnia Memory Association, Ylihärmä, Finland; 8 City of Seinäjoki, Finland; 9 Department of Psychology, University of Cambridge, Cambridge, UK; 10 Institute of Public Health and Clinical Nutrition, University of Eastern Finland, Kuopio, Finland; 11 Joint Municipal Authority for North Karelia Social and Health Services (Siun Sote), Joensuu, Finland; 12 Neuroepidemiology and Ageing Research Unit, School of Public Health, Imperial College London, London, UK; 13 Theme Aging, Karolinska University Hospital, Stockholm, Sweden; 14 Stockholms Sjukhem, Research & Development Unit, Stockholm, Sweden

## Abstract

**Background:**

The Finnish Intervention Study to Prevent Cognitive Impairment and Disability is a randomized controlled trial that has tested the efficacy of a multidomain intervention targeting modifiable risk factors to prevent cognitive impairment/dementia. A combination of healthy diet, physical, social and cognitive activity, and management of cardiovascular risks was shown to be an effective model to promote brain health among older people. The aim of this qualitative study was to explore healthcare professionals’ perceptions of facilitators and barriers to implementing this lifestyle programme into health care.

**Methods:**

Four semi-structured focus group interviews were conducted among healthcare professionals working in primary care and in non-governmental organizations (*N*=27). Participants were asked to discuss their perceptions of facilitators and barriers for implementing the multidomain intervention into clinical practice. Interviews were analyzed using content analysis.

**Results:**

Barriers and facilitators described by the healthcare professionals were related to infrastructure and resources, client’s personal characteristics and the lifestyle intervention itself. These main categories included several sub-categories related to knowledge, motivation, resources, individualization and collaboration. The interviewees pointed out that more education on dementia prevention is needed, the work should be coordinated efficiently, resources to provide preventive health care should be adequate and multiprofessional collaboration is needed.

**Conclusions:**

Transferring a lifestyle intervention from a trial-setting to real life requires knowledge about the factors that influence effective implementation. Identifying drivers and constraints of successful implementation helps to design and tailor future prevention programmes, increases motivation and adherence and supports system change.

## Introduction

The prevalence of dementia is drastically increasing with the population ageing, making it one of the greatest global public health challenges.^1^ There is no cure for dementia and only symptomatic treatment with limited efficacy is available. However, over the past decade, knowledge about lifestyle-related interventions promoting brain health has increased enormously, evoking growing interest in preventative approaches aiming to delay or prevent the disease onset. The Finnish Geriatric Intervention Study to Prevent Cognitive Impairment and Disability (FINGER) was the first large randomized controlled trial to show that modifying simultaneously several risk factors among older persons at risk for cognitive decline leads to improved cognitive performance, functional capacity and quality of life.^2–4^ The multidomain FINGER intervention consisted of physical and cognitive training, social activities, nutritional counselling and management of cardiovascular and metabolic risk factors. After 2 years, participants in the intervention group had over 30% lower risk for cognitive decline in comparison to the control group receiving only regular health advice.^3^ The intervention was effective and safe, and participants’ feedback was positive.^5^ FINGER serves as a pragmatic model for dementia prevention, and it is currently being adapted to different cultural and socioeconomic settings worldwide.^6^^,^^7^ Healthcare professionals and policy-makers have shown increasing interest in implementation. However, various challenges can arise when translating new evidence-based strategies into practice, leading to suboptimal guideline uptake and adherence.^8–10^

Knowledge of the best implementation practices of brain health interventions into real-life healthcare settings is limited. A recent scoping review mapped the published literature on primary care practitioners’ views on dementia risk reduction.^10^ The authors identified seven studies reporting factors that may affect effective implementation. The attitude towards dementia prevention seems to be positive, but there is uncertainty which risk factors to target, and the evidence supporting risk reduction is perceived as poor. Other immediate health issues have often higher priority than prevention. Healthcare practitioners also suffer from lack of time and continuity of clients’ care, and there are no practical tools to identify persons at-risk.^10^ Data included in the review was collected over 10 years ago when the knowledge of effective interventions was still scarce. Previous studies focussed on healthcare professionals’ current approaches and attitudes towards dementia prevention but did not address the challenges faced when implementing new evidence-based preventive actions.

In order to successfully implement new strategies into primary care and to support system change, it is important to better understand healthcare professionals’ current perceptions of dementia prevention, as well as their views on potential challenges related to implementation. Gaining understanding of the perceived barriers and facilitators help in developing practical models for efficient integration of the FINGER findings into primary care. The aim of this study was to explore healthcare professionals’ perceptions of facilitators and barriers to implementing FINGER multidomain lifestyle programme into clinical practice.

## Methods

### Study setting and participants

In this cross-sectional qualitative study, four focus group interviews with healthcare professionals from the public and third sector were conducted to explore the feasibility of incorporating the multidomain FINGER intervention^3^ into healthcare practice. First, two focus groups included primary care workers (total *N*=13) whose work involved health education and health promotion among older people. Third focus group was targeted at leaders or managers of health promotion work in primary care (*N*=8). The fourth focus group included healthcare professionals who work in non-governmental organizations or healthcare projects (*N*=6). Participants’ background factors were collected with a short questionnaire, and table 1 shows the participant characteristics. Mean age was 49 years and participants had on average 19 years of experience in working with older people. All participants gave their informed consent to participate in the study.

**Table 1:** Participant characteristics

	Focus groups 1 and 2^a^	Focus group 3	Focus group 4
	Primary care workers	Healthcare managers	Third sector and project workers
	*N*=13	*N*=8	*N*=6
Sex	9 (69)	8 (100)	4 (67)
Women men	4 (31)	0 (0)	2 (33)
Age in years	46 (11)	50 (9)	55 (16)
Work includes direct health education	11 (85)	5 (63)	4 (67)
Experience in working with older people in years	16 (13)	26 (12)	17 (12)

Data are shown as *N* (%) or mean (SD).

a Focus groups 1 and 2 included same type of primary care workers (nurses, physiotherapists and doctors).

### Data collection

Focus group interviews were conducted in the South Ostrobothnia area in Finland. Two researchers with qualitative research experience conducted the interviews; one was responsible for guiding the discussion and the other one took notes. A pre-defined semi‐structured interview sheet guided the interviews (table 2).

**Table 2:** Interview question guide

1. Attitudes and knowledge about dementia prevention	What do you know about dementia prevention?
Examples of questions	Do you believe that dementia can be prevented? Why yes or no?
2. Presentation of FINGER study results	What are your thoughts about the study?
Examples of questions	What are the strengths and weaknesses?
3. Implementation of FINGER model	Who can identify persons at risk for dementia and cognitive decline?
Examples of questions	What are the possibilities for dementia prevention and who is responsible? Where could a FINGER-type intervention take place? How should the intervention be adapted? How can you motivate older adults to change their lifestyles? What are the main barriers for effective implementation? Which factors facilitate the implementation? What should be taken into consideration when implementing a multidomain lifestyle intervention into clinical practice? What kind of practical tools are needed to help the implementation?

First, healthcare professionals were probed on their prior knowledge and perceptions of dementia prevention. After that, the interviewer presented briefly the FINGER trial and its main findings^3^^,^^4^ to stimulate the discussion. Finally, participants were asked to discuss the facilitators and barriers related to implementation of the FINGER model. Questions were open-ended, and participants were encouraged to freely develop the discussion, while the interviewer kept the conversation in-topic and probed topics that were not fully saturated. The interviews lasted about two hours each, and they were audiotape-recorded and transcribed verbatim.

### Data analysis

Data from all four interviews were analyzed as a whole, using content analysis with inductive category development. Analysis was carried out stepwise by two independent researchers (J.K. and A.R.). First, both researchers read all data repeatedly to obtain a sense of the whole. Second, transcripts were read word by word while highlighting the words and sentences describing facilitators and barriers to implementing the FINGER multidomain model into primary care. These highlighted meaning units included words, sentences or paragraphs that answered the research question. Meaning units were then sorted into sub-categories based on how they were related and linked.^11^ Finally, main categories were formed. Examples of quotations for each subcategory were selected and translated from Finnish to English. Two researchers (J.K. and A.R.) performed the analysis first independently and at the end discussed together and agreed on the final categories and results.

## Results

The following three main categories were formed to describe the different barriers and facilitators of implementing the FINGER intervention: (i) barriers and facilitators related to healthcare resources and infrastructure, (ii) person-related facilitators and barriers and (iii) barriers and facilitators related to the lifestyle intervention. The first and third main categories include eight sub-categories each; the second main category includes five sub-categories. All main categories and sub-categories are listed in table 3.

**Table 3:** Main categories and sub-categories related to facilitators and barriers identified by healthcare professionals

Main categories	Sub-categories
Barriers and facilitators related to healthcare resources and infrastructure	Mapping current resources, integrating dementia prevention into currently available healthcare services in cardiovascular disease and diabetes prevention Strengthen multiprofessional collaboration and communication between healthcare professionals and other stakeholders Shift the focus from disease treatment to prevention Lack of staff, time and financial resources Naming a coordinator or person in charge for promoting brain health Finding suitable locations for intervention activities, which are accessible for everyone Affordability of healthy lifestyle (e.g. food) Educating future professionals and policy-makers about prevention
Persons-related facilitators and barriers	Knowledge is an important motivating factor Finding time and own personal motivation for healthy lifestyle changes Changes should not be too big and hard to maintain, important to make changes that fit to personal daily routines Pain, diseases and disabilities Fear of dementia, stigma and negative attitude and preconceptions
Barriers and facilitators related to the lifestyle intervention	Introducing lifestyle changes gradually and taking small steps Maintaining motivation with personalized guidance and tailored advice Persons most in need/most likely to benefit are not easy to reach early enough Sufficient follow-up and continuity Social and peer support and group activities Positive feedback and encouragement Emphasis on well-being, not just disease prevention; holistic view when planning the intervention Using multiple channels including e-health technologies and internet to provide evidence-based information about prevention

### Barriers and facilitators related to healthcare resources and infrastructure

Integrating dementia prevention to current healthcare procedures was considered an effective way to increase knowledge of brain health among older adults. The interviewees thought that dementia prevention activities might not require additional resources if they are integrated into the work that is already ongoing in the field of cardiovascular and other non-communicable diseases.

‘I do not think we need additional resources, you can mention dementia prevention and make risk assessment when you are assessing also other risks.’

‘If you notice that a patient has elevated blood pressure, you should mention this [dementia risk] as well.’

‘We should do more comprehensive health assessment when the person comes to the appointment, not just focus on one single disease’.

Strengthening multiprofessional collaboration and early identification of persons at risk was also considered important. The interviewees referred to a current lack of collaboration between different healthcare sectors, which leads to different ways of working, lack of information and difficulties in implementing joint new models.

‘We do not get any information afterwards about how everything continues’.

‘Now everything is done separately, somebody takes care of this and somebody else takes care of that’.

‘Multiprofessional collaboration is the most important thing’.

Organizing comprehensive health assessments for older people was considered important, and doctors were seen important actors when implementing lifestyle interventions. Also, non-governmental organizations, especially local Alzheimer’s Associations, communities and occupational health care were seen very important collaborators.

‘Persons retiring should have comprehensive check-ups’.

‘Doctors should give information because people trust them like police’.

‘Local Alzheimer’s Association is very active, they hold different events and are also doing risk assessment.’

‘I think increasing knowledge in this field [dementia prevention] does not belong to just persons in the health care sector’.

‘We need services that are easily accessible, you should not need doctor’s prescription. Everybody should be able to join.’

‘Collaboration with other stakeholders is the solution. This is not somebody’s own thing; this belongs to everybody’.

Including additional education on brain health in curriculums for future healthcare professionals was suggested. The interviewees brought up that new scientific knowledge should be disseminated in schools and universities. In addition, healthcare professionals also pointed out that health promotion and dementia prevention are not seen as priorities at the political level and therefore educating policy-makers is also important.

‘This information [how to support brain health] should be incorporated in doctors’ training, as well as to education for physiotherapists, nurses, practical nurses, students in sport sciences, basically to everyone’.

‘There are communities where this work is well coordinated, but there are also communities where they say that this [the city hall] is not the place where this kind of work is done’.

‘Information on dementia prevention should be given to those who decide what kind of actions will be funded’.

However, healthcare professionals pointed out that there are problems, such as lack of staff, time and financial resources for prevention, and there is still more focus on disease treatment than prevention. The interviewees referred to their high work load. Busy working style does not allow implementation of new methods and innovations. In addition, lack of financial resources may prevent initiating new models and styles of working. The interviewees felt that it is particularly difficult to access nutritionist and physiotherapist services, and knowledge about dementia prevention may still be limited among healthcare professionals.

‘When your schedule is fully booked for the next two months, where do you put this kind of guidance, even though it is important’.

‘We already have so many other responsibilities’.

‘If you ask about nutrition or exercise, they [nurses] cannot answer’.

‘We should be able to give more dietary guidance, but with the current resources it is not possible. We would need more nutritionists or allocate more time to nurses for this’.

The healthcare professionals also felt that it is really important to identify a person who is in charge of coordinating the health promotion work. Otherwise, effective implementation is not possible.

‘There is no way this is going to be implemented into health care if nobody coordinates the work’.

‘Somebody needs to make a strategy and coordinate it’.

Providing facilities and finding locations for lifestyle intervention were considered important. External challenges, such as long distances, poor weather and lack of equipment may prevent older people from taking part in the intervention activities. The interviewees also pointed out that healthy food may be more expensive, which may be a barrier when trying to adopt a healthy lifestyle.

‘These practical things like poor weather, no equipment, overall tiredness’.

‘They don’t have money to buy healthy food, because nowadays healthy food is more expensive’.

‘Many people do not live in city centers or even close to any services’.

### Person-related facilitators and barriers

The second main category includes facilitators and barriers related to personal characteristics of older adults receiving the lifestyle intervention. The interviewees thought that people in general are interested in their own health and well-being, especially in terms of dementia prevention. They also pointed out that a healthcare professional cannot help a person to make lifestyle changes unless he/she is motivated to do so.

‘Regarding dementia prevention, I have noticed that information awakens people and motivates them’.

‘She/he [patient] must find his/her own motivation, then we can help’.

‘They [patients] want facts and results and if they are not getting these, it is not motivating’.

‘It is easy to ignore health advice if the information is too general. People think that this is not for them now, maybe in twenty years.’

‘They [patients] ask why they should make changes, they like their life when they do not have to do more than just stay on the couch.’

Individualized, personalized information and guidance and support in finding time for a new active lifestyle were considered important. Also, the lifestyle changes need to be small enough to be successfully incorporated into daily routines.

‘When thinking about risk factors, everybody does not have the same ones, so same information for all is not the best, we have to identify individual risk factors’.

‘People should notice small steps and take steps according to their own resources and abilities’.

‘We should take one step at the time, then give support when changes are happening’.

Among older adults, existing pain, pain induced by the intervention, diseases and disabilities affect the adherence to the interventions. Improving lifestyle may not be a priority if there are bigger challenges in patients’ lives.

‘One big barrier is the fear of pain. People think that pain is a sign of something you should not do.’

‘If you are physically ill or have pain, you do not have resources to take care of yourself.’

‘If a person has for example sleeping problems, there is no use to give dietary guidance. If the basic needs are not met, this does not work’.

Dementia is still surrounded by a stigma and associated with negative attitudes and preconceptions. This may prevent people from engaging in actions that aim to promote brain health. The clients may think that they are too old for lifestyle changes or have strong opinions on certain lifestyle behaviours. The interviewees also thought that for older people it may be difficult to know what information is relevant due to constant conflicting information from the media.

‘They do not get tested [memory tests], because they are so worried about the disease’.

‘We could take advantage of the fear, I mean by saying that this [dementia] is not something you just have to sit and wait for, instead you can do something to prevent it’.

‘They [patients] say that only “body builders” go to the gym, it is not a place for them.’

‘The biggest challenge is that there is a lot of different kind of information out there. One cannot say what is fact and what is fiction’.

### Barriers and facilitators related to the lifestyle intervention

The third main category reflects facilitators and barriers related to the relatively straining, time-consuming and long-term FINGER intervention. Participants pointed out that the implemented changes may be perceived as too big and challenging. People may, e.g. start exercising too intensively, get muscle pain and then stop. Thus, introducing lifestyle changes gradually and taking small steps is essential.

‘We should highlight small things that can be easily adapted to daily life’.

‘They [patients] may try to take too big steps and it is difficult to say that even small changes are enough.’

Also, if the guidance is not personalized, people will not get motivated. Therefore, healthcare professionals should focus on maintaining the clients’ motivation. This relates to a slow and easy start and personalized advice, positive attitude and encouragement. Healthcare professionals and older people should try to find out together the most important individual targets for changes. Providing evidence-based information and following up the progression were considered important. The interviewees also raised concerns that the people who are most in need of the intervention may not be reached.

‘We should aim to give “down-to-earth” type guidance’.

‘If you just send papers to people and ask them to do something, they just throw the papers away.’

‘They like to follow up the progress, maybe booklets or something they can show you’.

‘Those who would need the intervention the most do not actively seek help, and they are those who have many risk factors’.

Lack of social support was also seen as an important factor that may prevent participation in lifestyle interventions. Thus, group activities were strongly recommended. It is also important to see the person as a whole and emphasize overall well-being and not just disease prevention.

‘If there are changes [decrease] in social relationships, people may get depressed, feel useless and then they do not take care of themselves.’

‘When people feel that they belong to a meaningful group, then they accept more easily the suggested changes, whether it is physical activity or anything’.

‘We should see a person as a whole and think together what would be the first thing to change’.

‘It is not useful to talk about for example diet if a patient does not have any control over his/her life. We should focus on the bigger picture.’

The healthcare professionals also suggested that dementia prevention and promotion of healthy lifestyle could be implemented using e-health technologies and internet. Providing concrete, reliable, evidence-based information across multiple channels would increase the knowledge and thereby motivate older people to change lifestyle even without an intensive intervention.

‘Everybody is online, dementia prevention should be visible there, campaign or something’.

‘If the intervention and guidance could come for example from radio, people, even men, would be listening while doing something else’.

‘We would need a celebrity to promote this for example in TV during primetime’.

‘Virtual health promotion program’.

‘We already have online risk tests, maybe some of those could be done independently at home’.

## Discussion

To facilitate the effective implementation of an evidence-based FINGER lifestyle programme for older people, we carried out a qualitative study among healthcare professionals to assess facilitators and barriers to implementation. Barriers and facilitators related to infrastructure and resources, client’s personal characteristics and the lifestyle intervention itself were identified. Many of the factors discovered are consistent with the previous findings of studies investigating the implementation of dementia, diabetes and cardiovascular disease prevention programmes. Successful implementation is influenced by person-related factors comprising the characteristics, attitudes and behaviours of both the healthcare professional and the patient, as well as external factors that relate to the structure of the healthcare system, characteristics of the guidelines to be implemented and policies and messages enforced at the wider societal level.^10^^,^^12^ Structural barriers to prevention are lack of time and resources due to competing work demands and lack of specific funding for preventative activities.^10^^,^^12–16^ Challenges include lack of knowledge, awareness and/or skills of the healthcare professional,^17–19^ as well as negative attitudes and beliefs regarding prevention and its outcomes among both healthcare professionals and patients.^14^^,^^18^^,^^20^^,^^21^ In our study, also dementia-related stigma was identified as one of the barriers for prevention. On the other hand, shown also in our study continuing training opportunities,^22^^,^^23^ existence of appropriate information and support systems^23^^,^^24^ and clear staff roles and responsibilities^23^ are seen as key organizational facilitators to prevention. Easy-to-use, evidence-based and accessible guidelines,^15^^,^^25^^,^^26^ healthcare professionals’ intrinsic motivation towards prevention as well as its perceived importance^15^^,^^21^ and national health policies and strategies further play a role.^26^ Our results are largely in line with these previous findings.

It is obvious that all identified facilitators and barriers in this study are not equally doable and easy to deploy. Person-related barriers and facilitators and barriers and facilitators related to the lifestyle intervention are easier to address by providing practical tools, reliable information and education for healthcare practitioners. Barriers and facilitators that are related to infrastructure require system change, which is often difficult to achieve. The barriers related to infrastructure presented in this study can be considered chronic as they have been raised also in several previous reports.^27^ When planning the urgently needed changes, the effective drivers of successful implementation need to be taken into account. Successful change requires strong and skilled change leaders who are committed to drive the cultural transformation.^27^ Open dialogue and good relationships between leaders driving the change and change targets reduce the resistance and help to justify the need for change.^27^ Healthcare professionals need to learn to adopt inter-professional practices that improve teamwork and patient outcomes. The organizational structure, culture and processes that include information sharing have to support that.^28^ Passive dissemination of research evidence is usually ineffective, whereas educational visits, interactive educational meetings, audit and feedback support the system change.^29^^,^^30^ Observing contextual factors, involving relevant people, and knowing the characteristics of the patients will support transferring the research evidence to best practice.^27^^,^^30^

It should be noted that this study explored healthcare professionals’ perceptions of barriers and facilitators. It would be important to carry out a similar study also among older people who receive the intervention. We have previously reported that feedback from older persons participating in FINGER was very positive. Participants felt that the group activities motivated them to take part, the instructions were motivating, the intervention was individualized and their own wishes and opinions were taken into account.^5^ However, in-depth qualitative analyses targeting motivational factors, facilitators and barriers still need to be conducted.

This study revealed important facilitators and barriers that promote or hinder effective implementation of brain health interventions among older adults. The prevalence of dementia is expected to triple during the next three decades,^1^ but the prevention potential is high; theoretically up to 40% of cases could be delayed by modifying lifestyle.^31^ This sets urgent need to implement effective interventions especially to persons with established risk factors. Multidomain lifestyle programme that include physical activity, dietary guidance and management of vascular risk factors have beneficial effects on multiple outcomes, and are safe and well accepted by older persons. Table 4 summarizes the key considerations for successful implementation of multidomain lifestyle intervention to support brain health of older adults. Taking into consideration the facilitators and barriers identified in this study helps to implement the prevention model into health care.

**Table 4:** Key considerations for successful implementation of multidomain preventive interventions

Healthcare resources and infrastructure	Integrating dementia prevention into currently available healthcare services in cardiovascular disease and diabetes prevention
	Multiprofessional collaboration and communication
	Person responsible for coordination
	Education of healthcare professionals, policy-makers and stakeholders on dementia prevention
Person- and intervention-related factors	
	Evidence-based information about dementia prevention: knowledge increases motivation and reduces stigma surrounding dementia
	Personalized guidance and tailored advice
	Making small, gradual changes helps sustain the new habits
	Regular follow-up and positive feedback
	Emphasis on overall well-being instead of disease prevention
	Peer support, group activities and use of e-health tools
